# Characterization of Endophytic Fungi, *Acremonium* sp., from *Lilium davidii* and Analysis of Its Antifungal and Plant Growth-Promoting Effects

**DOI:** 10.1155/2021/9930210

**Published:** 2021-08-03

**Authors:** Mohammad Sayyar Khan, Junlian Gao, Iqbal Munir, Mingfang Zhang, Yixin Liu, The Su Moe, Jing Xue, Xiuhai Zhang

**Affiliations:** ^1^Beijing Agro-Biotechnology Research Center, Beijing Academy of Agriculture and Forestry Sciences, Beijing 100097, China; ^2^Genomics and Bioinformatics Division, Institute of Biotechnology and Genetic Engineering (IBGE), The University of Agriculture, Peshawar 25000 Khyber Pakhtunkhwa, Pakistan; ^3^Pharmaceutical Research Laboratory, Biotechnology Research Department, Ministry of Education, Mandalay Division, Kyaukse 05151, Myanmar

## Abstract

The present study was aimed at isolating endophytic fungi from the Asian culinary and medicinal plant *Lilium davidii* and analyzing its antifungal and plant growth-promoting effects. In this study, the fungal endophyte *Acremonium* sp. Ld-03 was isolated from the bulbs of *L. davidii* and identified through morphological and molecular analysis. The molecular and morphological analysis confirmed the endophytic fungal strain as *Acremonium* sp. Ld-03. Antifungal effects of Ld-03 were observed against *Fusarium oxysporum*, *Botrytis cinerea*, *Botryosphaeria dothidea*, and *Fusarium fujikuroi*. The highest growth inhibition, i.e., 78.39 ± 4.21%, was observed for *B. dothidea* followed by 56.68 ± 4.38%, 43.62 ± 3.81%, and 20.12 ± 2.45% for *B. cinerea*, *F. fujikuroi*, and *F. oxysporum*, respectively. Analysis of the ethyl acetate fraction through UHPLC-LTQ-IT-MS/MS revealed putative secondary metabolites which included xanthurenic acid, valyl aspartic acid, gancidin W, peptides, and cyclic dipeptides such as valylarginine, cyclo-[L-(4-hydroxy-Pro)-L-leu], cyclo(Pro-Phe), and (3S,6S)-3-benzyl-6-(4-hydroxybenzyl)piperazine-2,5-dione. Other metabolites included (S)-3-(4-hydroxyphenyl)-2-((S)-pyrrolidine-2-carboxamido)propanoic acid, dibutyl phthalate (DBP), 9-octadecenamide, D-erythro-C18-Sphingosine, N-palmitoyl sphinganine, and hydroxypalmitoyl sphinganine. The strain Ld-03 showed indole acetic acid (IAA) production with or without the application of exogenous tryptophan. The IAA ranged from 53.12 ± 3.20 *μ*g ml^−1^ to 167.71 ± 7.12 *μ*g ml^−1^ under different tryptophan concentrations. The strain was able to produce siderophore, and its production was significantly decreased with increasing Fe(III) citrate concentrations in the medium. The endophytic fungal strain also showed production of organic acids and phosphate solubilization activity. Plant growth-promoting effects of the strain were evaluated on *in vitro* seedling growth of *Allium tuberosum*. Application of 40% culture dilution resulted in a significant increase in root and shoot length, i.e., 24.03 ± 2.71 mm and 37.27 ± 1.86 mm, respectively, compared to nontreated control plants. The fungal endophyte Ld-03 demonstrated the potential of conferring disease resistance and plant growth promotion. Therefore, we conclude that the isolated *Acremonium* sp. Ld-03 should be further investigated before utilization as a biocontrol agent and plant growth stimulator.

## 1. Introduction

Endophytic fungi have long been recognized as biological agents that colonize the internal tissues of plants without causing any harm to the host plant [1]. It is estimated that more than one million endophytic fungi are prevalent and these have been identified in a large number of different plant species [2, 3]. These fungi were found in almost all parts of the plant such as root, leaves, stem, flowers, seed, and fruit. Endophytic fungi play significant contributions in agriculture development through their ability to act as biological control agents against a wide range of phytopathogens, insect pests, and nematodes [4]. These fungal endophytes mediate induced systemic resistance in plants that is considered an important mechanism for plant protection and disease management [5, 6].

Endophytic fungi are considered as the main source of bioactive compounds and secondary metabolites that have potential applications in various fields such as agriculture, pharmaceuticals, environmental cleaning, and the food industry [3, 7]. Some of the identified endophytic fungi produced bioactive compounds and metabolites, essential for plants to cope with biotic and abiotic stresses [8]. Bioactive compounds may also play an important role in plant protection against disease-causing pathogens and pests [9, 10]. Several studies have reported endophytic fungi with the ability to produce bioactive compounds [11], and also, some of these symbiotic endophytes promoted plant growth and productivity in several crop plants. However, the identified fungal strains with such beneficial properties are limited.

The endophytes' ability to promote plant growth might be associated with the production of growth hormones such as indole acetic acid (IAA). Previously, some endophytic fungi were reported as producers of indole acetic acid (IAA) [12, 13]. IAA is essential for plant growth and development from embryogenesis to senescence and mediates several developmental processes like axillary bud and flower formation and root development and improves several other processes [14]. In addition to IAA production, these fungal endophytes produce several other enzymes with important functions such as phosphate solubilization [15], pathogen resistance [16], and siderophore production [17]. Besides, endophytic fungi produce phytochemicals such as phenols and flavonoids that have potential applications in the medicinal and agrochemical industries. These phytochemicals with antioxidant properties play important roles in protecting host plants against abiotic stresses [18–20].

Members of the genus *Acremonium* have long been recognized as biological agents that confer resistance to plants against insect pests, nematodes, and environmental stresses like drought stress [21, 22]. Several *Acremonium* endophytes have been reported from different perennial grasses where these endophytes enhanced plant resistance against insect pests [23]. The antifungal role of *Acremonium* species has also been established since long ago. Tomato and flax plants inoculated with *A. ochraceum* and *A. strictum* reduced the wilt symptoms due to *F. oxysporum* infection [21]. *Acremonium* sp. (ENF 31) isolated from maize showed resistance against phytopathogens, *viz*, *Pythium ultimum*, *Sclerotium oryzae*, *Rhizoctonia solani*, and *Pyricularia oryzae* [4]. Endophytic fungi produce a wide variety of bioactive and secondary metabolites with potential medicinal applications. Based on chemical structures and biosynthetic pathways, these bioactive compounds have been divided into four groups, including alkaloids, nonribosomal peptides, polyketides, and terpenes [24]. Several studies have confirmed the isolation of antifungal compounds from endophytic *Acremonium* strains isolated from different plants. In one study, the antifungal compounds 1-heptacosanol and 1-nonadecane were isolated from the biocontrol fungus *Acremonium* sp. (MPHSS-2.1) through GC-MS chromatography [25]. Many species of the *Acremonium* genus have been identified as producers of useful metabolites. Cephalosporins, which belong to the *β*-lactam class of antibiotics, were derived from *A. strictum*. The anti-inflammatory sesquiterpenoids were derived from a sponge-derived *Acremonium* sp. in Korea [26]. A mangrove-derived *Acremonium* sp. was reported to produce phthalide and isocoumarin derivatives [27]. Besides, *Acremonium cellulolyticus* is known to be a potential producer of cellulase [28, 29]. Pyrrocidines A and B are polyketide-amino acid-derived antibiotics that were derived from *Acremonium zeae*, an endophyte of maize kernels [30]. Pyrrocidines exhibited significant antifungal activity against mycotoxin-producing *Aspergillus flavus* and *Fusarium verticillioides*. Pyrrolopyrazine alkaloid (also known as loline alkaloids) was characterized from *Acremonium lolii*, an endophytic fungus of perennial ryegrass (*Lolium perenne* L.) [31]. Leucinostatin A, an oligopeptide with anticancer, phytotoxic, and antifungal properties, was detected from *Acremonium* sp., an endophyte of *Taxus baccata* [32].

*L. davidii* var. unicolor is one of the species in the genus *Lilium* of the family Liliaceae. Plants of this species hold immense economic importance due to their applications in food, gardening, and pharmaceutical industries [33]. This species is a rich reservoir of protein, mineral nutrients, sugars, and vitamins [34]. The flowers of this species are highly nutritive and thus are used as a new food source in China [35]. Several studies have revealed the medical and health benefits of the bulbs of *L. davidii*. Health effects may include the production of antioxidants, relieving cough, and anxiety symptoms; immunity-boosting; and blocking tumor development [36]. No study has been conducted until this date that could describe the association of *L. davidii* with beneficial endophytic fungi and its role in plant growth and disease resistance.

In the present study, a fungal endophyte Ld-03, identified as *Acremonium* sp., was isolated from the bulbs of *L. davidii*. We assumed that this endophyte could confer antifungal activity against disease-causing pathogens and could facilitate plant growth through the production of plant growth-promoting enzymes, and secondary metabolites.

## 2. Materials and Methods

### 2.1. Sample Sterilization and Endophyte Isolation

For endophytic fungal isolation and other tests, we used the methods described by Khan et al. [37]. The bulb samples of *L. davidii* were used for endophytic fungi isolation. Bulbs were collected from the experimental fields of the Beijing Agriculture Biotechnology Research Center, Academy of Agriculture and Forestry Sciences, China. After storage at 4°C, fresh and healthy-looking bulbs were selected for fungal isolation. Sample preparation and endophytic fungal isolation were carried out according to the previously described protocol [38]. Bulbs were rinsed with tap water until all dust particles and impurities were removed. The bulbs were then peeled off aseptically, and the outermost layers were removed. Bulb samples were then treated with 70% (*v*/*v*) ethanol for 1 min followed by immersion in 10% (concentration of active chlorine) NaClO solution for 20 min. The samples were then washed with sterile distilled water three times. After surface sterilization, the outer layer on both sides of each bulb portion was removed. Samples were then cut into pieces of approximately 1 cm × 1 cm and inoculated on potato dextrose agar (PDA) plates. The PDA plates were incubated at 24°C ± 1°C until fungal growth appeared on the bulb portions. After one to two weeks of incubation, the fungal mycelia were aseptically inoculated onto fresh PDA plates and PD broth and were incubated at 24°C ± 1°C until pure cultures were obtained. The broth culture was stored as glycerol stock at -80°C.

### 2.2. Identification of Fungal Strains

The endophytic fungal strain termed as Ld-03 was cultured on PDB and incubated at 24°C ± 1°C until mycelial growth appears and spread to the plate. The fungal strain was then investigated for morphological and microscopic observation. Mycelial and conidial structures were observed using a light microscope. For scanning electron microscopic observations, 1 ml fungal culture was centrifuged at 8000 rpm for 5 min. The supernatant was discarded, and the cell pellet was washed thrice with 1 ml 0.2 M phosphate buffer (PBS) (pH 7.2-7.4). The pellet was then fixed with 2.5% glutaraldehyde for 3 hr. After fixation, the pellet was washed twice with PBS followed by rinsing with pure water. The pellet was then dehydrated by the concentration gradient of 30%, 50%, 70%, 80%, and 90% of ethanol for 15 min at each step and then dehydrated twice for 15 min in 100% ethanol. Fungal mycelial and conidial structures were observed using the SU8010 field-emission scanning electron microscope (SEM, Hitachi, Japan).

For molecular analysis, the endophytic fungal strain was inoculated in PDB at 24°C ± 1°C for 48 h in a shaker at 150 rpm. The fungal culture was then centrifuged at 4000 rpm, room temperature for 10 min. The supernatant was discarded, and the cell pellet was used for genomic DNA extraction using the fungal Genomic DNA Isolation Kit (SolarBio) following the manufacturer's protocols. Molecular identification was conducted through the amplification of the ITS rDNA sequences. About 544 bp sequence was amplified from genomic DNA using primers ITS1F and ITS4R specific for the rDNA genes. A 25 *μ*l PCR reaction contained 1 *μ*l (0.5-10.0 ng) of template DNA, 0.2 *μ*M each primers ITS1F and ITS4R, 200 *μ*M of each dNTP, 10x buffer, 2 mM MgSO_4_, and 1 U High-Fidelity KOD Taq DNA Polymerase. The cycle parameters were as follows: initial denaturation at 95°C for 5 min; 30 cycles of denaturation for 30 s at 94°C, annealing for 30 sec at 52°C, and extension for 1 min at 68°C; and a final overall extension for 7 min at 68°C. The PCR product was purified using the QIAquick PCR Purification Kit (Qiagen, Hilden, Germany) and was then sequenced. Sequences were BLAST searched against homologous fungal ITS rDNA sequences using NCBI. The determined sequence was aligned using CLUSTAL W, and phylogenetic trees were constructed based on neighbor-joining (NJ) and maximum likelihood (ML) algorithms using the MEGA 7 software [39]. The nucleotide sequence was then submitted to GenBank under accession number MN393594.

### 2.3. Antifungal Activity

Antifungal activities of the isolated fungal endophyte were determined against four strains of pathogenic fungi, i.e., *F. oxysporum*, *B. cinerea*, *B. dothidea*, and *F. fujikuroi*. These four pathogenic strains were selected based on an *in vitro* pathogenicity test showing the potential of causing infection in *L. davidii*. The antifungal bioassay was conducted using the dual culture method. A 5 mm plug of the pathogenic fungi was placed in the middle of the PDA plate. Two plugs of 5 mm each of the endophytic fungal strain Ld-03 were placed about 3 cm away from the pathogenic plug. Plates containing pathogenic plugs at the center without endophytic fungi were used as controls. The plates were incubated at 24°C ± 1°C. Plates were observed for fungal growth regularly, and the progression of the fungal growth was monitored. The zone of inhibition of fungal growth was measured after the fungal mycelia in the control plates reached the edges of the plates. Growth inhibition of the fungal pathogen was calculated using the formula: %of growth inhibition = [(*C* − *T*)/*C*] × 100, where *C* is the radial growth of the test pathogen in the control plates (mm) and *T* is the radial growth of the test pathogen in the test plates (mm). The experiment was repeated thrice.

### 2.4. Antifungal Assay of Ethyl Acetate Fraction

The ethyl acetate fraction of the isolated endophytic fungal strain Ld-03 was used to test the antifungal activity against pathogenic fungi: *F. oxysporum*, *B. cinerea*, and *F. fujikuroi*. The antifungal susceptibility test was determined by the disc diffusion assay [40]. Sterile discs, 6 mm in diameter, impregnated with 20 *μ*l of ethyl acetate extract of the endophytic fungal strain Ld-03 were dried in the laminar hood. These discs were then placed in the center of the PDA plates. Two pugs (6 mm in dia) of the pathogenic fungi were placed about 3 cm away from the disc in both directions. Plates with disc impregnated with 10% DMSO were used as the negative control. The plates were incubated at 24°C ± 1°C, and the diameter of the zone of inhibition was recorded.

### 2.5. Ethyl Acetate Extraction of Fungal Secondary Metabolites

The extraction of fungal secondary metabolites was done by the solvent partition method. The fungal isolate Ld-03 was grown in potato dextrose broth at 24°C for 7-8 days. After the incubation period, the broth cultures were taken out and filtered through sterile filter paper to remove the mycelia mats. An equal volume of the filtrate and ethyl acetate was taken into the separating funnel and shaken for the complete extraction. The solvent phase that contains secondary metabolites was separated from the aqueous phase, and the solvent was evaporated to dryness to yield the crude extracts. The crude extract (20 mg) was redissolved in 1 ml of 70% methanol. 500 *μ*l of the dissolved extract was filtered through a 0.2 *μ*m syringe filter before ultrahigh performance liquid chromatography LTQ XL linear ion trap mass spectrometry/mass spectrometry (UHPLC-LTQ-XL-IT-MS/MS) analysis.

### 2.6. UHPLC-LTQ-XL-IT-MS/MS Analysis for Secondary Metabolite Profiling

UHPLC-LTQ-IT-MS/MS analysis was performed using the method partially adapted from Lee et al. [41]. The Thermo Fisher Scientific LTQ XL linear ion trap mass spectrometry consisted of an electrospray interface (Thermo Fisher Scientific, San José, CA, USA) coupled with a DIONEXUltiMate 3000 RS Pump, RS Autosampler, RS Column Compartment (Dionex Corporation, Sunnyvale, CA, USA) was used for secondary metabolite profiling of the fungal extracts. The sample was separated on a Thermo Scientific Hypersil GOLD C18 column with a 1.9 *μ*m particle size. The mobile phase consisted of A (0.1% (*v*/*v*) formic acid in water) and B (0.1% (*v*/*v*) formic acid in acetonitrile), and the gradient conditions were increased from 10% to 100% of solvent B. Scanning was set to start after 1 min to the source. Solvent gradient time was set over 19 min and reequilibrated to the initial condition for 4 min by setting the divert valve to waste. The flow rate was set at 0.3 ml/min, and the injection volume was 10 *μ*l. The temperature of the column during measurement was maintained at 35°C. The ion trap was performed in positive and full-scan ion modes within a range of 150–1000 *m*/*z*. The operating parameters were as follows: source voltage, ±5 kV, capillary voltage, 39 V; capillary temperature, 275°C, auxiliary gas flow rate 10−20 arbitrary units, sheath gas flow rate 40−50 arbitrary units, spray voltage 4.5 kV. The tandem MS (MS/MS) analysis was performed by scan-type turbo data-dependent scanning (DDS) under the same conditions used for MS scanning for the six most intense ions using the *N*th order double play mode. MS data was acquired by Xcalibur software, Thermo Fisher Scientific.

### 2.7. Putative Identification of Secondary Metabolites

Putative identification of secondary metabolites was done using molecular networking workflow from the GNPS website (https://gnps.ucsd.edu) [42]. Raw LC-MS files were converted into mzXML using ProteoWizard3.0.19140 [43], and the mzXML file was uploaded to GNPS. A molecular network was created using the default parameters. The spectra in the network were then searched against the GNPS spectral libraries. The library spectra were filtered in the same manner as the input data. All matches kept between network spectra and library spectra were required to have a score above 0.7 and at least 6 matched peaks.

### 2.8. Indole Acetic Acid (IAA) Detection

Indole acetic acid (IAA) in the isolated fungal strain Ld-03 was detected according to the method of [44] with minor modifications [45]. Potato dextrose broth (PDB) was prepared with 10% tartaric acid to prevent any bacterial growth. The PDB was provided four different concentrations of exogenous tryptophan, i.e., 0 mg ml^−1^, 1 mg ml^−1^, 2 mg ml^−1^, and 4 mg ml^−1^. A fungal disc (5 mm in dia) was inoculated into 10 ml PDB, supplemented with the above-mentioned tryptophan concentrations in 50 ml flasks. Samples were incubated for one week at 25°C ± 1°C under dark conditions on a shaker with shaking of 120 rpm. Fungal cultures were then centrifuged at 12000 rpm for 10 min at 4°C. After centrifugation, 1 ml supernatant from each sample was mixed with 2 ml Salkowski reagent (98 ml 35% HClO_4_, 2 ml 0.5 M FeCl_3_) and was incubated in the dark for 30 min. The change of color from yellow to pink was considered as positive for IAA production. The IAA contents were quantitatively measured by taking absorbance at 530 nm in a spectrophotometer. The IAA quantities in samples were measured based on a standard curve of known values.

### 2.9. Siderophore Detection

The ability of the endophytic fungal strain Ld-03 to produce siderophore was tested by Chrome Azurol S (CAS) assay, a method developed by Schwyn and Neilands [46]. The fungal mycelia were cultured in a PDB medium for 7-10 days at 24°C and 120 rpm shaking. After incubation, a fungal sample was added to Minimal Media 9 (MM9) with CAS solution and supplemented with different iron concentrations, i.e., 0 *μ*M, 0.25 *μ*M, 2 *μ*M, and 4 *μ*M Fe(III) citrate. Samples were again incubated at 24°C for 6 days with 120 rpm shaking. After incubation, 100 *μ*l of the blue Chromium Azurol S (CAS) reagent was added to samples followed by incubation for 4 h at room temperature. The change of color from blue to orange/yellow was considered as positive. Siderophore concentrations in all samples were further measured at 630 nm. The siderophore quantities were measured as % of siderophore units by the formula: %of siderophore units = Ar − As/Ar∗100, where “Ar” is the absorbance of reference (CAS reagent) and “As” is the absorbance of the sample at 630 nm. Qualitative detection was done on Chrome Azurol S (CAS) blue agar. The isolated strain Ld-03 was inoculated on CAS agar plates and incubated at 24°C under dark condition for two weeks. The appearance of yellow/orange hallows around the colonies confirmed siderophore production. All assays were carried out in triplicates.

### 2.10. Organic Acid Production

Organic acids were detected through a method by Cunningham and Kuiack [47] with modifications. About 50 *μ*l of the fungal suspension in 10 mM MgSO_4_ was inoculated in NBRIP liquid media. Samples were incubated at 24°C for 5 days and 150 rpm shaking. The organic acids were then detected by adding 100 *μ*l of 0.1% alizarine red S pH indicator. The samples were incubated at room temperature for 15 min. The samples with yellow color were positive, while pink color was negative for organic acid production.

### 2.11. Phosphate Solubilization Assay

The endophytic strain Ld-03 was grown on a PDA medium at 25°C for 7-9 days. The phosphate solubilizing ability of the strain was detected on Pikovskaya's (PKV) agar as previously described [48, 49]. After inoculation on the media, the strain was grown at 28°C for 48 h. After the incubation period, the phosphate solubilization activity was observed as clearing zones around the colonies.

### 2.12. Plant Growth-Promoting Effect of Fermentation Broth

The effect of fermentation broth was tested on the *in vitro* growth of *A. tuberosum* Rottl.ex Spreng. The isolated fungal strain Ld-03 was cultured in PDB for ten days at 25 ± 2°C with 120 rpm shaking. The culture was then centrifuged at 5000 rpm for 10 min at 4°C. The supernatant was filtered through Whatman filter paper grade 1 : 11 *μ*m (medium flow filter paper). The flow-through was used as the fungal elicitor and was diluted with sterile water to 10%, 40%, and 70%. The *A. tuberosum* seeds were sterilized with 75% ethanol for 1 min followed by rinsing in ddH_2_O. Seeds were then immersed in 3% sodium dichloroisocyanurate for 20 min and finally washed with sterile water. Eight seeds were placed in Petri dishes (*Φ* = 9 cm) with a filter paper. About 3 ml of different concentrations of the filtrate was added to each Petri dish. The Petri plates with sterile water and PDB were used as the control plates. Petri dishes were incubated at 25/18 ± 2°C (day/night) for 5 days. After incubation, the root and shoot elongations of seedlings were recorded. Each treatment was repeated five times.

### 2.13. Statistical Analysis

Data analysis was performed using analysis of variance (ANOVA). Statistically significant differences between the two groups were identified using the Student *t*-test at *P* ≤ 0.05.

## 3. Results

**Figure 1:**
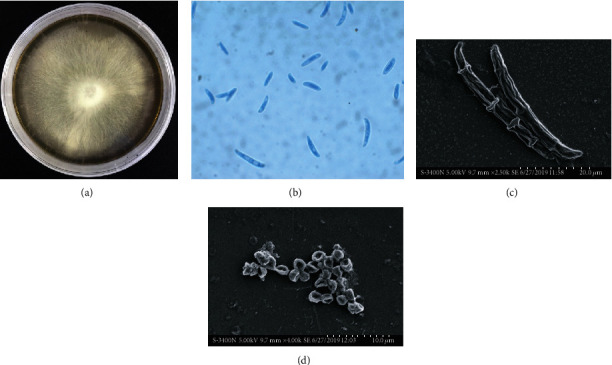
Fungal growth and microscopic observation of the isolated fungal strain Ld-03. Colony cultures on potato dextrose agar incubated at 25 ± 1°C for 7-10 days (a). Light micrograph showing fungal conidia, stained with lactophenol cotton blue (b). Bar indicates 20 *μ*m. Scanning electron microscopic (SEM) analysis of the endophytic *Acremonium* sp. isolated from *L. davidii*. Structures of mycelia and spores (c, d).

**Figure 2:**
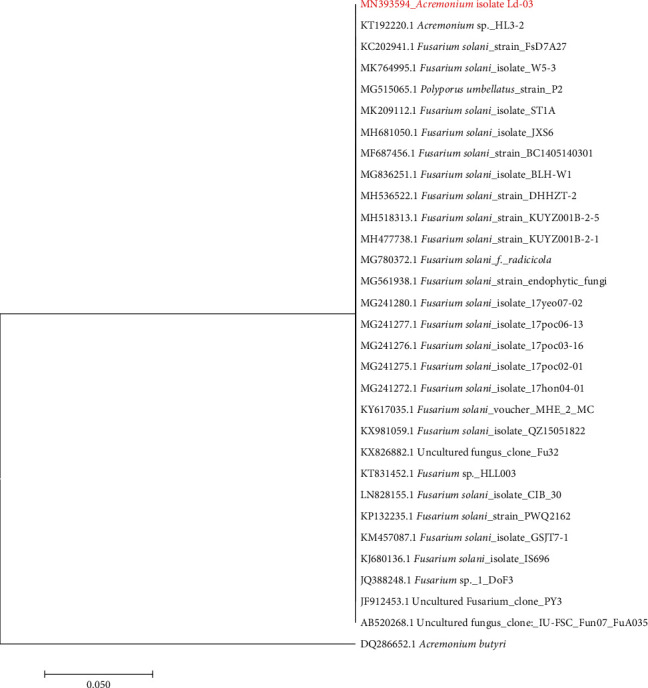
Maximum likelihood phylogenetic tree based on the 16S rRNA ITS gene sequences of fungal strain Ld-03 and related strains. The evolutionary history was inferred by using the maximum likelihood method based on the Tamura-Nei model (1993). *Acremonium butyri*, DQ286652.1, was used as an out-group. Bar 0.050 substitutions per nucleotide position. Evolutionary analyses were conducted in MEGA7 (Tamura et al. [39]).

Endophytic fungal strain Ld-03 identified as *Acremonium* sp. was isolated from the bulbs of *L. davidii*. The isolated strain was identified using microscopic and molecular techniques. The Ld-03 strain formed compact and moist colonies with loose and cottony hyphae with white color on PDA plates at 24°C ± 1°C (Figure 1(a)). The fungus produced cylindrical-shaped conidia (Figure 1(b)). Scanning electron microscopy revealed structures of mycelia, conidia, and spores, typical of the *Acremonium* sp. (Figures 1(c) and 1(d)). The ITS sequence obtained through PCR with specific primers was submitted to the NCBI GenBank database. The sequence was matched with homologous sequences through the BLAST search for final identification. Sequences with high similarity were selected and aligned using “CLUSTAL W” followed by the construction of a phylogenetic tree based on the maximum likelihood method in MEGA 7.0 (Figure 2). The BLAST results revealed that the 544 bp long ITS rDNA gene sequence indicated that the strain is closely related to *Acremonium* sp. The isolated Ld-03 strain showed a 100% similarity with *Acremonium* sp. strain HL3-2 (KT192220.1). The ITS rDNA gene sequence of Ld-03 was submitted to GenBank under accession number MN393594.1.

**Figure 3:**
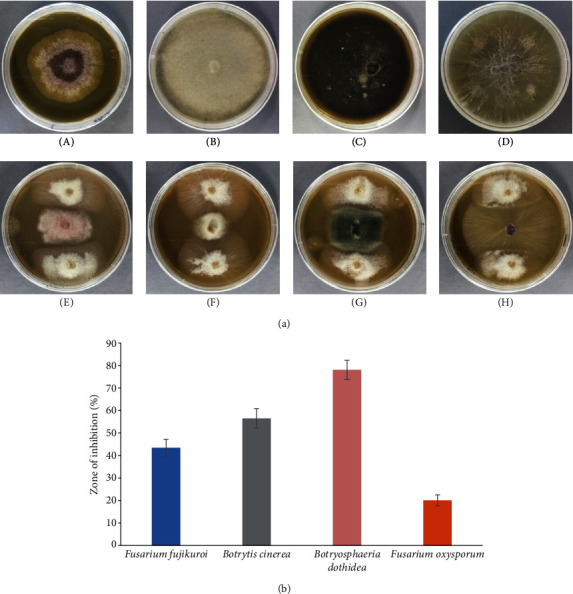
Antifungal effects of the endophytic *Acremonium* sp. against four pathogenic strains using dual culture assay. (a) A 5 mm plug of pathogenic fungi was cultured in the middle of the PDA plate surrounded by two plugs of the endophytic fungi. Plates (A), (B), (C), and (D) are controls of *F. fujikuroi*, *B. cinerea*, *B. dothidea*, and *F. oxysporum*, respectively. PDA plates (E), (F), (G), and (H) contain dual cultures. (b) Antifungal activities were measured as the size of the zones of inhibition (ZI) of the pathogenic fungi. Zones of inhibitions were measured after two weeks of fungal growth. Means were averages ± standard deviation. The experiment was repeated thrice with *n* = 5.

The isolated endophytic Ld-03 strain was tested for its antifungal/antiproliferative ability against four different pathogenic fungal strains like *F. oxysporum*, *B. cinerea*, *B. dothidea*, and *F. fujikuroi*. These pathogenic strains were previously tested in an *in vitro* study for their pathogenicity potential and disease-causing ability in bulbs of *L. davidii*. All pathogenic fungal strains revealed disease symptoms in bulbs of *L. davidii* (Fig. S1). The endophytic fungal strain Ld-03 showed moderate to high antifungal activity against the tested fungal pathogens. The antifungal results showed that the isolated fungal strain Ld-03 restricted the proliferation of the fungal pathogens as revealed in the dual culture assay (Figure 3(a)). The highest percentage of growth inhibition, i.e., 78.39 ± 4.21%, was observed for Ld-03 against *B. dothidea* followed by 56.68 ± 4.38%, 43.62 ± 3.81%, and 20.12 ± 2.45% against *B. cinerea*, *F. fujikuroi*, and *F. oxysporum*, respectively (Figure 3(b)).

The antifungal property of the isolated strain Ld-03 was further tested by the disc diffusion method using the ethyl acetate fraction. The effect of ethyl acetate fraction was evaluated on the growth of *F. fujikuroi, F. oxysporum*, and *B. cinerea* on PDA plates incubated at 24°C ± 1°C. Ethyl acetate fraction showed inhibitory effects on the growth of all tested fungal pathogens (Fig. S2). Regular observation of the growth and proliferation of pathogenic fungi revealed an inhibitory effect of the extract. The ethyl acetate fraction slowed down the growth of fungal pathogens as indicated by comparison with the control plates. The inhibitory zones of the fungal pathogens around the ethyl acetate fraction were recorded after nine days of culturing. The inhibitory zones of *F. fujikuroi*, *F. oxysporum*, and *B. cinerea* were 33 ± 1.3 mm, 22 ± 0.8 mm, and 30 ± 1.4 mm, respectively.

As the isolated fungal strain Ld-03 showed high antagonistic effects against different fungal pathogens, it was speculated that the strain could be a source of bioactive secondary metabolites. Therefore, the ethyl acetate fraction of Ld-03 was analyzed through UHPLC-LTQ-IT-MS/MS. Identification of putative secondary metabolites was done using molecular networking workflow from the GNPS website. A molecular network was created using the default parameters. The spectra in the network were then searched against the GNPS spectral libraries. The total ion current chromatogram of the endophytic fungal strain Ld-03 showed peaks of numerous compounds in the ethyl acetate fraction (Fig. S3).

About 15 putative compounds belonging to different groups were identified from the endophytic fungal strain Ld-03 (Table 1). Some of the compounds identified were previously characterized and exhibited antifungal and antibacterial properties. Some of the prominent bioactive compounds identified from the strain Ld-03 were xanthurenic acid (a quinoline monocarboxylic acid that has a role as an iron chelator) and valyl aspartic acid. Gancidin W is a potential low-toxicity antimalarial agent that possesses essential bacterial, antifungal, antiviral, and anticancer activities. Cyclic dipeptides such as cyclo-[L-(4-hydroxy-Pro)-L-leu], cyclo(Pro-Phe), (3S,6S)-3-benzyl-6-(4-hydroxybenzyl)piperazine-2,5-dione (cyclodipeptide), and valylarginine (a dipeptide composed of valine and arginine) were identified. Other metabolites included (S)-3-(4-hydroxyphenyl)-2-((S)-pyrrolidine-2-carboxamido)propanoic acid and dibutyl phthalate (DBP). DBP is a member of the group of chemicals commonly known as phthalates that have previously been reported with antifungal and antibacterial activities. Other bioactive compounds detected were 9-octadecenamide, (Z) (anti-inflammatory and antimicrobial activity), D-erythro-C18-Sphingosine (sphingolipids with antimicrobial activities), N-palmitoyl sphinganine and hydroxypalmitoyl sphinganine (sphingosine), and 2-(21-amino-3,20-dihydroxydocosan-2-yl)oxy-6-(hydroxymethyl)oxane-3,4,5-triol(aminoglycolipid).

**Figure 4:**
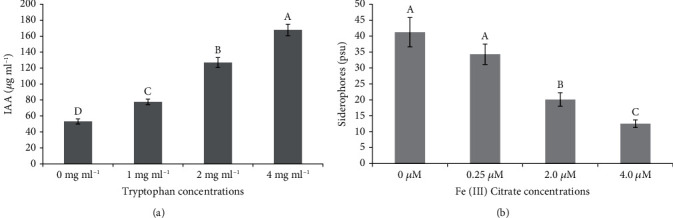
Indole acetic acid (IAA) and siderophore quantification in Ld-03. The IAA quantities were measured at various tryptophan concentrations (a). Siderophore was quantified at various Fe(III) citrate concentrations (b). Data are averages ± SD (*n* = 6). Bars with the same letters did not differ significantly at a significant level (*P* ≤ 0.05).

Qualitative detection of IAA was confirmed through a change of color from yellow to pink (Fig. S4c). The IAA quantities were further recorded in the presence and absence of exogenous tryptophan (Figure 4(a)). The strain Ld-03 produced 53.12 ± 3.20 *μ*g ml^−1^ IAA without exogenous tryptophan application (0 mg ml^−1^) in the culture medium. When tryptophan was added to the medium, the IAA content increased, indicating that tryptophan could accelerate the IAA production. The addition of 1 mg ml^−1^ tryptophan to the culture medium increased the IAA production to 77.67 ± 3.42 *μ*g ml^−1^. However, further increasing the tryptophan concentration in the medium had a much higher impact on the ability of the strain to produce IAA. The strain Ld-03 accumulated 126.83 ± 6.32 and 167.71 ± 7.12 *μ*g ml^−1^ IAA at 2 mg ml^−1^ and 4 mg ml^−1^ exogenous tryptophan concentrations, respectively. It indicated that the increasing tryptophan had no negative effect on IAA production; rather, it highly increased its production.

The isolated endophytic fungal strain Ld-03 was tested for its potential to produce siderophore (Figure 4(b)). The production of siderophore was investigated in the absence and presence of an iron source in the medium. The strain was cultured in the liquid 284 medium supplemented with different Fe(III) citrate concentrations, i.e., 0 *μ*M, 0.25 *μ*M, 2.0 *μ*M, and 4.0 *μ*M. The strain Ld-03 showed high siderophore production when cultured in a medium without Fe(III) citrate. Siderophore production was recorded as 41.2 ± 4.6 (psu) in the culture medium without the addition of Fe(III) citrate. However, the production of the siderophore in the culture medium declined significantly (*P* ≤ 0.05) with the addition of various quantities of Fe(III) citrate. Siderophore production was slightly decreased, i.e., 34.3 ± 3.2 (psu) at 0.25 *μ*M Fe(III) citrate in the culture medium. However, a significant reduction in the production of the siderophore was observed when Fe(III) citrate content was raised to 2.0 *μ*M and 4.0 *μ*M. The strain produced 20.1 ± 2.1 and 12.5 ± 1.2 (psu) siderophore. These results suggested that the strain Ld-03 can produce siderophore under limited iron availability in the medium.

A qualitative test further revealed the production of siderophore by forming a yellow/orange halo surrounding the fungal colonies. On Chrome Azurol S (CAS) agar plates, the Ld-03 produced a visible yellow/orange halo that averaged about 1.5 cm in radius (Fig. S4a,b). The ability of siderophore production is an indication that the strain Ld-03 may accelerate and promote plant growth partly by providing the rarely available iron sources in the soil.

A qualitative test revealed the production of organic acids in the endophytic strain Ld-03 through a change of color from pink to orange (Fig. S4d). The phosphate solubilization activity was observed through a clearing zone surrounding the fungal mycelia growth on Pikovskaya's agar plates.

The effects of various concentrations of fermentation broths were investigated on the *in vitro* shoot and root growth of *A. tuberosum* (Table 2). Compared to the control condition (water and medium), it was found that the fermentation broths promoted the seedling growth of *A. tuberosum* at different concentrations. Particularly, 40% diluted broth promoted seedling growth compared to nontreated plants (Fig. S5). Shoot length increased significantly when the fermentation broths were diluted to 10%, 40%, and 70%. However, maximum shoot length (37.27 ± 1.86 mm) was recorded at 40% diluted fermentation broth. Similar was the case with the root growth as fermentation broths increased root growth compared to the control treatment. Significantly higher root growth, i.e., 24.03 ± 2.71 mm, was observed at 40% diluted fermentation broth. Therefore, the fermentation broth from the endophytic fungal strain Ld-03 promoted the growth of *A. tuberosum*.

## 4. Discussion

The genus *Acremonium* contains many species; most are saprophytic being isolated from dead plant material and soil. Several species are recognized as opportunistic pathogens of man and animals, causing mycetoma, mycotic keratitis, and onychomycosis. Recently, several *Acremonium*-like species recognized as opportunistic pathogens have been transferred to other genera [50, 51]. This genus is morphologically simple in terms of taxonomy; therefore, classification at the species level is difficult. The general morphological features include septate hyphae with simple, tapered, lateral phialides produced singly or in groups and unicellular, globose-to-cylindrical conidia, which are mostly aggregated in slimy heads at the apex of the phialide [52]. Nonetheless, their taxonomy has not been firmly resolved due to the absence of clear-cut morphological differences at the species level and the absence of reliable sequences in public databases [53, 54]. Morphologically, *Acremonium* and *Fusarium* species are very similar and may be confused with each other but *Fusarium* usually grows faster and has colonies with a characteristic fluffy appearance. In our study, the isolated endophytic fungal strain Ld-03 showed the highest similarity with *Acremonium* sp. HAL 3-2, followed by *Fusarium solani* strains as indicated by the phylogenetic tree.

*Acremonium* species are frequently identified as endophytes of many plant species [55, 56]. Endophytic association of *Acremonium* sp. has been reported from a wide range of hosts like Festuca grass of North America [57] tropical forage grass, *Brachiaria brizantha* [58], *Taxus globosa* [59], and *Sesbania grandiflora* [60]. *Acremonium* sp. has also been isolated as an endophyte from the roots of the traditional Chinese medicinal herb, *Macleaya cordata*, and its extracts exhibited strong inhibition on test bacteria [61]. Petroski et al. [62] have shown that species of *Acremonium* increased alkaloid production in its host, *Stipa robusta*. Strobel et al. [32] reported antifungal activity by *Acremonium* sp. isolated from *Taxus buccata*. Prathyusha et al. [63] reported isolated fungal endophyte *Acremonium sclerotigenum* from *Terminalia bellirica*, an important medicinal plant of tropical deciduous forests of Telangana state, India. This isolate showed not only the antibacterial nature of the crude extract but also the ability to inhibit plant pathogenic fungi like *F. oxysporum* and *C. dematium*. *Acremonium* species along with mycelia sterilia dominated the endophytic fungi of Egyptian medicinal plants. Isolates of *A. strictum* from different hosts have shown a significant difference in biological activity [56]. *Acremonium* sp. (MPM-2.1) isolated from *Cicer arietinum* exhibited antiplant pathogenic activity against phytopathogens such as *Sclerotinia sclerotiorum*, *B. cinerea*, *F. oxysporum*, and *Rhizoctonia solani* [25]. In this paper, we report, for the first time, the endophytic association of *Acremonium* from *L. davidii*, an important plant with aesthetic, medicinal, and edible properties. The isolated endophytic fungal strain Ld-03 exhibited antifungal/antiproliferative activity against four different fungal pathogens, like *F. oxysporum*, *B. cinerea*, *B. dothidea*, *and F. fujikuroi.* The ethyl acetate extract of this isolate also showed inhibitory effects against the tested phytopathogens. The tested phytopathogens revealed disease symptoms in the *in vitro* pathogenicity test. Therefore, the broad-spectrum antifungal activities of both the isolated Ld-03 strain and its ethyl acetate fraction suggest the potential of this endophytic strain for bioactive compounds involved in the plant endophytic relationship.

Endophytic fungi in association with plants have been known to produce diverse secondary metabolites with antipathogenic and plant growth-promoting effects [64]. As previously discussed, the endophytic *Acremonium* isolates in different studies showed the presence of bioactive compounds and secondary metabolites with antipathogenic properties. Likewise, in the present study, the isolated *Acremonium* strain Ld-03 showed antifungal activities against different phytopathogens. Several bioactive compounds and secondary metabolites belonging to different groups were putatively identified from the ethyl acetate extract. Prominent bioactive compounds identified in the present study included gancidin W, cyclic dipeptides such as cyclo-[L-(4-hydroxy-Pro)-L-leu], cyclo(Pro-Phe), (3S,6S)-3-benzyl-6-(4-hydroxybenzyl) piperazine-2,5-dione (cyclodipeptide), dibutyl phthalate, 9-octadecenamide, (Z), and sphingosines. Most of the identified compounds in the present study were previously isolated from different endophytic fungi, and these compounds showed broad-spectrum antimicrobial activities. Gancidin W, a nitrogenous bioactive compound, was isolated from the marine *Streptomyces* sp. VITLGK012 showed broad-spectrum antibacterial potential [65]. Cyclic dipeptides cyclo(L-Phe–L-Pro) and cyclo(L-Phe–trans-4-OH-L-Pro) were identified from the lactic acid bacteria, *Lactobacillus plantarum* MiLAB 393 isolated from the grass silage [66]. The identified compounds showed activities against food- and feed-borne filamentous fungi (*Fusarium sporotrichioides* and *Aspergillus fumigatus*) and yeast (*Kluyveromyces marxianus*). The bioactive compound dibutyl phthalate was previously recovered by ethyl acetate from the fermentation broth of the soil isolate, *Streptomyces albidoflavus* 321.2 [67]. The identified bioactive compound showed strong activity against gram-positive and gram-negative bacteria, as well as unicellular and filamentous fungi. Further, Ahsan et al. [68] reported the identification of dibutyl phthalate from *Streptomyces* strain KX852460 that showed antifungal activity against *R. solani* AG-3 KX852461, the causal agent of target spot disease in tobacco leaf. The bioactive compound, 9-octadecenamide, (Z) identified in the present study was previously reported. Mohammed et al. [69] reported 9-octadecenamide along with other compounds from the medicinal plant, pomegranate (*Punica granatum* L.). Plant extracts exhibited antibacterial and antifungal activities. Bharose and Gajera [70] identified 9-octadecenamide, (Z) in *Bacillus subtilis* that was highly active against the aflatoxin-producing *Aspergillus*. In the present study, bioactive compounds belonging to sphingosines and sphingolipids were identified in the isolated strain Ld-03. Previous studies have demonstrated that several sphingoid bases and fatty acids act as antibacterial agents against a variety of gram-positive and gram-negative bacteria [71]. Becam et al. [72] demonstrated the bactericidal activity of sphingolipids, including the sphingoid base sphingosine against pathogenic *Neisseria*. The antimicrobial activity of sphingolipids is thought to be a result of their ability to interact with the microbial cell wall [73].

Plant growth-promoting traits are distinguishing features of endophytic microbes associated with plants. Fungal endophytes produce several enzymes such as indole acetic acid, gibberellins, siderophore, and organic acids that are useful for the growth and productivity of plants. Indole acetic acid production is an important trait that directly stimulates plant growth. Several *Acremonium* species isolated from different plants were found with indole acetic acid production that showed positive effects on plant growth. Previously, *Acremonium* endophyte isolated from tall fescue produced IAA under in vitro culturing conditions [74]. Plant inoculation with the isolated endophyte resulted in morphological alterations and increased plant growth. However, the correlation between IAA production and altered growth expressions upon endophyte inoculation of the tall fescue was termed inconclusive. IAA and siderophore production was detected in the endophytic fungi *A. sclerotigenum* isolated from *Terminalia bellirica* [63]. *Acremonium* sp. (ENF 31) isolated from maize was reported to produce defensive enzymes that potentially resulted in inhibition of the growth of phytopathogens like *Pythium ultimum*, *Sclerotium oryzae*, *Rhizoctonia solani*, and *Pyricularia oryzae* [4]. The isolated strain also produced IAA and siderophore. In the present study, the isolated strain Ld-03 produced IAA without the application of tryptophan in the medium. However, when different doses of tryptophan were applied, the IAA production increased and there was a positive correlation between tryptophan and the IAA contents. The utilization of tryptophan is an important characteristic of plant growth-promoting endophytes. Plant roots secrete tryptophan, and the endophytes and actinomycetes in the rhizosphere can utilize tryptophan to produce IAA, which, in turn, promotes plant growth [75]. Moreover, the strain Ld-03 produced siderophore as indicated by qualitative and quantitative tests. This is evident from the fact that even trace amounts of iron present in the soil may indirectly promote plant growth. Several studies have shown that microbial secretions in the rhizosphere can interact with the iron present in the soil [76, 77]. The fungal strain Ld-03 showed production of organic acids and phosphate solubilization activity. Both these traits are important plant growth-promoting traits and were previously reported in several fungal strains isolated from various plant hosts [78, 79].

Both these plant growth-promoting traits of the isolated endophyte might be responsible for the improved root and shoot growth of *A. tuberosum* under *in vitro* conditions. Previous studies had shown that plant roots and seedlings were used as indicators of plant growth [80]. Therefore, we examined the effect of diluted fermentation broths of the Ld-03 on the seedling growth of *A. tuberosum*. Fermentation broth with 40% dilution significantly increased the seedling growth of *A. tuberosum*. This is an indication that the fermentation broth of Ld-03 contained plant growth-promoting agents like IAA, siderophore, and other secondary metabolites which promoted the growth of *A. tuberosum*. The ability of the isolated *Acremonium* sp. Ld-03 to produce IAA, siderophore, and bioactive secondary metabolites gives this endophyte an added advantage to be a potential biocontrol agent of plant pathogens and a plant growth stimulator.

## 5. Conclusion

This is the first report of isolation and assessment of endophytic fungi from *L. davidii*. The isolated endophytic fungal *Acremonium* sp. Ld-03 exhibited antifungal and plant growth-promoting effects. The presence of several already reported bioactive secondary metabolites in the ethyl acetate fraction of Ld-03 is an indication that this strain possesses considerable antifungal and plant growth-promoting potential. Further studies should be conducted before utilization of Ld-03 as an agent to confer disease resistance and plant growth promotion in future sustainable agriculture.

## Figures and Tables

**Table 1:** Overview of the putative compounds detected in the ethyl acetate extract of endophytic fungal strain Ld-03.

Compound name	*m*/*z* measured	Library *m*/*z*	Molecular formula	Adduct	GNPS score	GNPS library ID	CAS no.
Xanthurenic acid	205.0	206.0	C_10_H_7_NO_4_	M+	0.71	CCMSLIB00000221272	59-00-7
Gancidin W	212.1	211.1	C_11_H_18_N_2_O_2_	M + H	0.80	CCMSLIB00000081180	N/A
Cyclo-[L-(4-hydroxy-Pro)-L-leu]	227.1	227.1	C_11_H_18_N_2_O_3_	M + H	0.94	CCMSLIB00000081185	N/A
Cyclo(Pro-Phe)	245.1	245.0	C_14_H_16_N_2_O_2_	M + H	0.83	CCMSLIB00003134825	511126
(S)-3-(4-Hydroxyphenyl)-2-((S)-pyrrolidine-2-carboxamido)Propanoic acid	261.1	261.1	C_14_H_18_N_2_O_4_	M + H-H_2_O	0.95	CCMSLIB00003138923	19786368
Valyl arginine	275.3	274.1	C_11_H_23_N_5_O_3_	M + H	0.73	CCMSLIB00003139299	N/A
Dibutyl phthalate	279.1	279.1	C_16_H_22_O_4_	M + H	0.95	CCMSLIB00003135352	84742
9-Octadecenamide, (Z)	282.3	282.3	C_18_H_35_NO	M + H	0.93	CCMSLIB00003138290	301020
D-erythro-C18-Sphingosine	301.2	300.2	C_18_H_37_NO_2_	M + H	0.86	CCMSLIB00003135697	123784
(3S,6S)-3-Benzyl-6-(4-hydroxybenzyl)piperazine-2,5-dione	311.2	311.0	C_18_H_18_N_2_O_3_	M + H	0.74	CCMSLIB00000211287	5147-17-1
N-Palmitoyl sphinganine	541.0	539.5	C_34_H_70_NO_3_	M + H	0.72	CCMSLIB00003106239	N/A
Hydroxypalmitoyl sphinganine	557.0	555.5	C_34_H_70_NO_4_	M + H	0.71	CCMSLIB00003089836	N/A
2-(21-Amino-3,20-dihydroxydocosan-2-yl)oxy-6-(hydroxymethyl)oxane-3,4,5-triol	558.5	558.4	C_28_H_57_NO_8_	[M + Na]+	0.79	CCMSLIB00004709211	N/A

**Table 2:** The effect of fermentation broth of Ld-03 on *in vitro* growth of *A. tuberosum*.

Treatments	Shoot length (mm)	Root length (mm)
Control (water + PDB)	22.81 ± 2.27^c^	15.61 ± 1.74^b^
10%	32.44 ± 2.49^ab^	17.77 ± 1.95^b^
40%	37.27 ± 1.86^a^	24.03 ± 2.71^a^
70%	31.39 ± 2.09^ab^	17.79 ± 2.11^b^

Means are averages ± standard deviations (SD). Values in a column with different letters are significantly different by the Student *t*-test at *P* ≤ 0.05.

## Data Availability

The data used to support the findings of this study are available from the corresponding author upon request.

## Supplementary Materials

Supplementary 1 Figure S1: *in vitro* pathogenicity test of the fungal pathogens against *L. davidii*. Bulbs of *L. davidii* were inoculated with fungal pathogens by putting mycelia on the wounded surface of the bulbs. Plate (a) is the control without fungal inoculation, while plates (b), (c), (d), and (e) show bulb inoculation with *B. dothidea*, *F. fujikuroi*, *F. oxysporum*, and *B. cinerea*, respectively.

Supplementary 2 Figure S2: antifungal effect of the ethyl acetate fraction of endophytic *Acremonium* sp. against pathogenic strains using disc diffusion assay. A 6 mm disc having 20 *μ*l of the ethyl acetate fraction was placed in the middle of the PDA plate surrounded by two plugs of the pathogenic strains. Plates (a), (c), and (e) are the controls of *F. fujikuroi*, *F. oxysporum*, and *B. cinerea*, respectively. In the control plates, the discs were impregnated with 10% DMSO. Zones of inhibitions were measured after nine days of fungal growth.

Supplementary 3 Figure S3: the total ion current (TIC) chromatogram of the endophytic fungal strain Ld-03.

Supplementary 4 Figure S4: qualitative analysis of plant growth-promoting traits. Siderophore was detected on CAS blue agar plates. Fungal growth of the isolated strain Ld-03 on simple PDA media (a). Siderophore production was detected as a yellow/orange halo surrounding the fungal mycelia growth on CAS agar plates (b). The production of IAA in Ld-03 was observed as a change of color from yellow to pink (c). The strain showed organic acid production (d).

Supplementary 5 Figure S5: the effect of 40% diluted fermentation broth of Ld-03 on the root and shoot growth of *A. tuberosum*. CK means blank control.
