# Supplementary figures and images for: Characterization of Endophytic Fungi, Acremonium sp., from Lilium davidii and Analysis of Its Antifungal and Plant Growth-Promoting Effects
Source: Biomed Res Int. 2021 Aug 3;2021:9930210. doi: 10.1155/2021/9930210 (PMC8358427; doi:10.1155/2021/9930210)

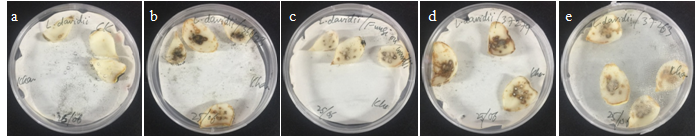

Supplement: Supplementary 1 — Figure S1: in vitro pathogenicity test of the fungal pathogens against L. davidii. Bulbs of L. davidii were inoculated with fungal pathogens by putting mycelia on the wounded surface of the bulbs. Plate (a) is the control without fungal inoculation, while plates (b), (c), (d), and (e) show bulb inoculation with B. dothidea, F. fujikuroi, F. oxysporum, and B. cinerea, respectively. [file 9930210.f1.png]

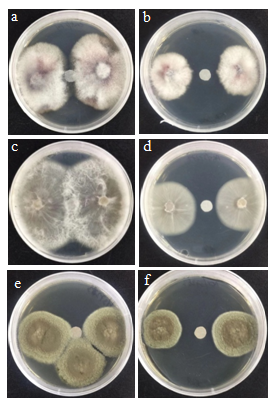

Supplement: Supplementary 2 — Figure S2: antifungal effect of the ethyl acetate fraction of endophytic Acremonium sp. against pathogenic strains using disc diffusion assay. A 6 mm disc having 20 μl of the ethyl acetate fraction was placed in the middle of the PDA plate surrounded by two plugs of the pathogenic strains. Plates (a), (c), and (e) are the controls of F. fujikuroi, F. oxysporum, and B. cinerea, respectively. In the control plates, the discs were impregnated with 10% DMSO. Zones of inhibitions were measured after nine days of fungal growth. [file 9930210.f2.png]

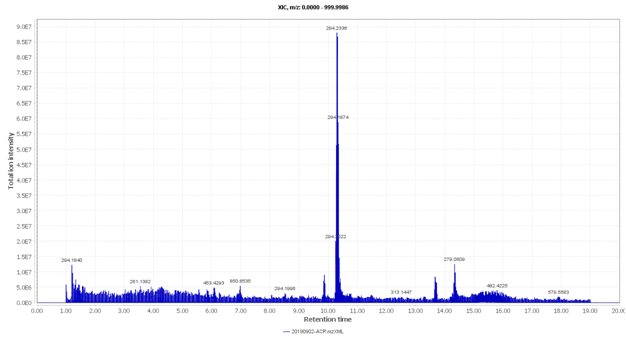

Supplement: Supplementary 3 — Figure S3: the total ion current (TIC) chromatogram of the endophytic fungal strain Ld-03. [file 9930210.f3.png]

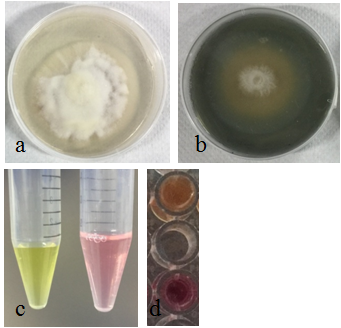

Supplement: Supplementary 4 — Figure S4: qualitative analysis of plant growth-promoting traits. Siderophore was detected on CAS blue agar plates. Fungal growth of the isolated strain Ld-03 on simple PDA media (a). Siderophore production was detected as a yellow/orange halo surrounding the fungal mycelia growth on CAS agar plates (b). The production of IAA in Ld-03 was observed as a change of color from yellow to pink (c). The strain showed organic acid production (d). [file 9930210.f4.png]

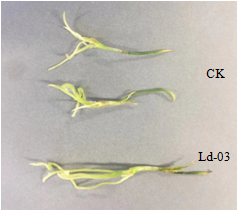

Supplement: Supplementary 5 — Figure S5: the effect of 40% diluted fermentation broth of Ld-03 on the root and shoot growth of A. tuberosum. CK means blank control. [file 9930210.f5.png]
